# Composition of Coloured Gastric Residuals in Extremely Preterm Infants-A Nested Prospective Observational Study

**DOI:** 10.3390/nu12092585

**Published:** 2020-08-26

**Authors:** Gayatri Athalye-Jape, Megan Nettleton, Ching-Tat Lai, Elizabeth Nathan, Donna Geddes, Karen Simmer, Sanjay Patole

**Affiliations:** 1 Neonatal Directorate, King Edward Memorial Hospital, Perth 6008, Western Australia, Australia; gayatri.jape@health.wa.gov.au (G.A.-J.); megan.nettleton@health.wa.gov.au (M.N.); karen.simmer@uwa.edu.au (K.S.); 2 Centre for Neonatal Research and Education and Division of Paediatrics, Medical School, University of Western Australia, Perth 6008, Western Australia, Australia; 3 School of Molecular Sciences, University of Western Australia, Perth 6009, Western Australia, Australia; ching-tat.lai@uwa.edu.au (C.-T.L.); donna.geddes@uwa.edu.au (D.G.); 4 Department of Biostatistics, Women and Infants Research Foundation, Perth 6008, Western Australia, Australia; liz.nathan@uwa.edu.au; 5 Division of Obstetrics and Gynaecology, University of Western Australia, Perth 6008, Western Australia, Australia

**Keywords:** preterm, green residuals, nutrition

## Abstract

Green gastric residuals (GR) are often considered as a sign of feed intolerance and discarded in preterm infants. Probiotics are known to enhance feed tolerance in preterm infants. To assess the composition (primary outcome) and volume of discarded green GRs, and feeding outcomes in extremely preterm (EP) infants in a probiotic trial, composition of pale and dark green GRs in the first two weeks of life from EP infants (<28 weeks) in a randomized controlled trial (RCT: SiMPro) of single vs. three-strain probiotics was assessed. Feeding outcomes included time to full feeds (TFF: 150 mL/kg/day) and duration of parenteral nutrition (PN). EP infants given placebo in our previous probiotic RCT served as the reference group. Analysis involved linear regression modelling with clustered standard errors for repeated measurements. GRs of 74/103 from 39 SiMPro infants (18: single-strain, 21: three-strain) were analyzed. Bile acid content was higher but statistically insignificant (825.79 vs. 338.1 µmol/L; *p* = 0.12) in dark vs. pale green GRs. Mean (95% confidence interval) fat, nitrogen, and carbohydrate loss in GRs over the study period was 0.02 g (0.01–0.03), 0.011 g (0.009–0.013), and 0.05 g (0.04–0.06), respectively. Overall, SiMPro infants had shorter median TFF (10 vs. 14 days, *p* = 0.02) and duration of PN (10 vs. 16 days, *p* = 0.022) compared with control group infants. Z scores for growth parameters at discharge were comparable. Discarding dark green GRs meant higher loss of bile acids during early enteral nutrition in EP infants. Probiotic supplementation was associated with reduced TFF and duration of PN.

## 1. Introduction

Optimizing early nutrition is a priority in preterm infants, as suboptimal nutrient intake in the first few weeks of life is associated with adverse effects on long-term growth and neurodevelopment [1,2,3,4,5,6,7]. Early introduction of feeding is an important strategy in this context, especially in extremely preterm (EP) infants. However, grading up of milk feedings to an optimal volume is affected by decisions based on routine monitoring and interpretation of gastric residual (GR) volume and colour [8,9,10], a practice that lacks robust high-quality evidence [11,12,13]. Parker et al. have [14,15] reported that discrepancies in the definition and interpretation of abnormal GRs affect clinical practice and make research in this field challenging [13,14,15]. Their randomized controlled trial (RCT) showed that infants who did not receive pre-feed GR assessment achieved significantly better enteral nutrition, weight gain, and a shorter hospital stay. The risk for necrotizing enterocolitis (NEC), death, late onset sepsis (LOS), and ventilator associated pneumonia was not increased [15].

Bile stained, especially dark green GRs, are often interpreted as signs of feed intolerance, and discarded [16,17]. However, there is no clear evidence to support this practice. Bile acids have a physiological role in regulation of gut motility and hepatic lipid, glucose, and energy homeostasis [18,19,20,21,22]. Furthermore, bile acids have anti-inflammatory effects [23,24], and may have an important role in regulating intestinal and hepatic components of innate immunity [25,26]. Therefore, discarding dark green GRs containing bile acids is a potential barrier to optimizing enteral nutrition in early postnatal life in EP infants.

Considering the clinical significance of the issue, we aimed to assess the composition (bile and other nutrients) of bilious GRs in EP infants enrolled in our RCT, comparing single vs. three-strain probiotic supplementation. Our hypothesis was that dark green GRs will have higher bile acid content compared to pale green GRs.

Probiotics are known to reduce the risk of NEC, LOS, mortality, and feeding intolerance in preterm infants [27]. Improved gut motility is an important mechanism for benefits of probiotics in reducing feed intolerance in preterm infants [28,29]. We therefore aimed to assess whether the volume of GRs and the time to full feeds (TFF) was reduced in probiotic supplemented vs. unsupplemented EP infants (secondary hypothesis).

## 2. Materials and Methods

### 2.1. Design, Set-Up and Ethics Approval

This prospective study was nested within our double-blind RCT (SiMPro, ANZCTR CTRN12615000940572) of single (Bifidobacterium/B. *breve* M-16 V) vs. multi-strain (a combination of *B. breve* M-16V, *B. longum subsp. infantis* M-63, *B. longum subsp. longum* BB536) probiotics in EP infants admitted between September 2015 and October 2016.

### 2.2. Participants

*Eligibility criteria*: (1) Gestation <28 weeks; (2) recruited in SiMPro trial and ready to commence on feeds; (3) informed written parental consent.

*Exclusion criteria*: (1) Major congenital malformations; (2) chromosomal aberrations; (3) on feed for ≥24 h.

### 2.3. Outcomes

Bile (µmole/L) and other nutrient content carbohydrates (g/L), protein (g/L), and fat (g/L) of pale and dark green GRs in EP infants in the single vs. multi-strain probiotic arms of SiMPro trial.

Volume of GRs and TFF in probiotic supplemented vs. unsupplemented infants: Infants in both arms of the SiMPro trial were supplemented with a probiotic (single or three-strain). Prior to the SiMPro trial, we routinely provided probiotic supplementation to all preterm infants <34 weeks [30]. We hence selected probiotic unsupplemented EP infants (born before GA 28 weeks) from the placebo arm of our previous probiotic trial (PANTS) as the “control” group [31].

Separate approval was obtained from the institutional ethics committee (Number: 26737) for retrospective collection of data from the PANTS trial [31].

Other outcomes: These included feeding volumes (FV), NEC ≥ stage II, LOS, and growth parameters at discharge.

### 2.4. GR Samples and Data

(1) Discarded GRs from EP infants in SiMPro trial were collected in the first two weeks of life and stored at −20 degrees Celsius before analysis. (2) Data on GR volume (GRV), color (green/hemorrhagic), and GRV as percentage of FV was collected prospectively for the first 28 days in EP infants in the SiMPro trial, and retrospectively from EP infants for a similar period in the placebo arm of the PANTS trial [31].

### 2.5. Preparation of GR Samples

The GR samples stored at −20 °C were thawed at 22 °C for 30 min and transferred to a pre-weighted tube (15 mL Falcon polypropylene tube). The filled tubes were re-weighed to determine the net volume of transferred GR.

### 2.6. Analysis of Nutrient Content of GRs

GR samples were homogenized with the Sonics VCX 130 ultrasonic processor. The pH was measured using the pH meter (Orion Stara Series, Thermo Scientific, Waltham, MA, USA) with the manufacturer’s two-point standard calibration method (pH 4–7). Fifty µL of the sample was pipetted into a well of a 96-well plate. Each sample was processed in duplicate. The plate was inserted into a UV/Vis spectrometer (Enspire, Perkin Elmer, Waltham, MA, USA) to scan from 300–1000 nm to determine the max absorption wavelength of each sample. The osmolality was measured in duplicate by freezing point osmometry using the Fiske Model 110 Osmometer (Advanced Instruments Inc., Norwood, MA, USA). The total nitrogen content was measured using the modified Kjeldahl method [32]. The total sugar content was determined by the sulfuric acid method [33]. Total bile acid content was measured using the total bile acid kit (DZ042A-K, Diazyme Laboratories, Poway, CA, USA). The fat content was measured by the esterified fatty acid method [34].

### 2.7. Feeding Protocol for EP Infants during Probiotic Trials

A standardized protocol was followed for feeding EP infants during SiMPro and PANTS trials. Briefly, (1) expressed breastmilk was preferred for feeding. (2) Feeding was initiated as early as possible as two hourly boluses through the orogastric tube. (3) An infant was “ready to commence on feeds” once clinical stability was achieved based on the following criteria: (a) No/minimal respiratory assistance (e.g., mean airway pressure: ~8 cm H_2_O, continuous positive airway pressure (CPAP) support ~5–6 cm H_2_O and oxygen ~30%); (b) blood pressure within normal range without cardiovascular support; (c) no hemodynamically significant patent ductus arteriosus (PDA); (d) no sepsis or sepsis treated with antibiotics for at least for 48 h, and no respiratory or haemodynamic compromise. (4) Continuous feeds were used for infants weighing <750 g with persistent intolerance to bolus feeding. Infants on continuous milk feeding (CMF) had the orogastric tube aspirated every 4 hours, primarily to check tube position. If the GR volume was >30% of total feeds over the previous 4 h or more than the hourly feeding volume, the GR was returned if it was milky and the next feed was withheld. Feeds were continued in the presence of isolated green GRs with normal clinical examination. Feeds were withheld for persistent or worsening dark green GRs. The residuals were discarded in such cases. (5) Frequency of feeding was changed to every 3 h after reaching full feeds (150 mL/kg/day). (6) Depending on gestation and growth status at birth, feeds were started at 5 or 10 mL/kg/day, increased to 15–20 mL/kg/day, and subsequently graded up by 7.5–10 mL/kg every 8–12 h till reaching a volume of 150 mL/kg/day. TFF was the time in days from starting minimal enteral feeds to achieving a feeding volume of 150 mL/kg/day. The maximum daily total milk volume was 170 mL/kg/day, increased from 150 mL/kg/day in increments of 10 mL/kg/day at 48 h intervals. (7) Feeding was withheld in the presence of pre-feed GRV >50% of the previous two feedings, significant bile- or blood-stained GR, or abdominal distension, and during red cell transfusions. (8) There was no change in the use of pasteurized donor human milk during both trials. (9) Interpretation of GRs: This was based on a color-coded chart for assessing the color of GRs (www.adhb.govt.nz/newborn/Guidelines/Nutrition/WithholdingFeeds) [17]. Both pale green (wasabi and lime) and dark green (avocado and spinach) GRs were discarded, whereas milky, lemon, and mustard colored GRs were re-fed.

### 2.8. Statistical Methods

Continuous data were summarized using medians, interquartile ranges (IQR), and ranges (R), and categorical data were summarized using frequency distributions.

#### 2.8.1. Nutrient Content Analysis

Measurements of pH, osmolality, total nitrogen, carbohydrates (CHO), bile acid, and fat content were compared between pale and dark green GRs using linear regression models with clustered standard errors on subjects to account for multiple measurements.

#### 2.8.2. GRV Analysis

Univariate continuous demographic and clinical characteristics were compared between control, single-, and three-strain groups using the Kruskal-Wallis test and categorical outcomes compared using the Chi-square test or exact methods. When the overall result was statistically significant, pairwise comparisons were made with the Bonferroni correction to maintain an overall alpha error rate of 0.05. Linear regression modelling was used to assess the influence of probiotic treatment on GR volume as a percentage of feed volume in 24 h over the duration of time in days from the age at commencing minimal enteral feeds to the age at reaching 150 mL/kg day, and on the time in days until full feeds were established. Intrauterine growth restriction (IUGR) and CRIB scores were adjusted for in the models due to the baseline imbalance between groups. A subgroup analysis excluding IUGR infants was also performed to assess whether the results remained consistent when this large baseline imbalance was removed.

Outcome measurements in both analyses were transformed to the natural logarithm when necessary to satisfy assumptions of residual distributional normality. Mean estimates were back-transformed and reported on the original scale as geometric means and 95% confidence intervals (CI). All tests were two-sided, and *p*-values < 0.05 were considered statistically significant. SPSS statistical software version 22.0 (IBM Corp, Armonk, NY, USA) and Stata version 16 (Statacorp, College Station, TX, USA) statistical software were used for data analysis.

## 3. Results

A total of 29 infants from the placebo arm of the PANTS trial (controls), and 154 infants from the SiMPro trial (single strain: 75 and three-strain: 79) were included. Control infants were more likely to have IUGR than those treated with probiotics (control: 44.8% vs. single-strain: 6.7% vs. three-strain: 5.1%; *p* < 0.001). They also had lower median CRIB scores than the single-strain probiotic group (10 vs. 12; *p* = 0.009 with Bonferroni correction) (Table 1). However, CRIB scores between infants in the two arms of the SiMPro trial were comparable. Other demographic characteristics were similar (Table 1). SiMPro trial infants commenced probiotics significantly earlier compared to placebo commencement age in control group infants (median age: 3 days vs. 7 days; *p* < 0.001; Table 2).

### 3.1. GRs in SiMPro Trial Infants

Of the GR samples, 74/103 had adequate volume for analysis. They were obtained from 39 infants with a median gestational age (GA) and birth weight (BW) of 26.5 weeks and 810 g, respectively. The median (IQR) samples per infant were 1 (1–2), respectively. There were 18 (46.2%) infants in the single-strain, and 21 infants (53.8%) in the three-strain group. Their median (IQR) GA and BW were 26.3 (24.7–27.3) weeks and 870 (708–1010) g, and 26.6 (25.0–27.3) weeks and 920 (750–1070) g, respectively (Table 1).

### 3.2. Outcomes

(1) Bile and other nutrient compositions of pale vs. dark green GRs: (a) Comparison irrespective of the allocation to single- vs. three-strain probiotics: Estimated means, differences, and 95% CI for each bile content measure compared between pale (used as reference) and dark green colour grades are shown in Table 3. Bile acid content was higher in dark vs. pale green GRs (825.79 vs. 338.1 µmol/L). Mean pH, osmolality, nitrogen, fat, and carbohydrate content were comparable. (b) Comparison between single- and three-strain probiotic arms of SiMPro trial: There were no differences in the bile and other nutrient content between pale and dark green GRs when single- and three-strain SiMPro data were analysed. Descriptive summaries of the raw values of GR contents (volume, pH, osmolality, nitrogen, carbohydrate, bile acid, and fat) for the total sample and stratified by colour grade (pale green: *n* = 15 vs. dark green: *n* = 59) and by SiMPro group (single- or three-strain) are summarised in Supplementary Table S1a,b.

(2) *Other nutrient loss in coloured GRs*: Loss of fat, nitrogen, and carbohydrates was calculated for 74 GR samples over the two-week study period (mean estimates and 95% CI): fat: 0.02 (0.01–0.03) g, nitrogen: 0.011 (0.009–0.013) g, and carbohydrate: 0.05 (0.04–0.06) g, respectively (Table 3).

(3) *GRV, TFF, and other outcomes*: These included 29 control group infants, and 154 SiMPro trial infants (single-strain: 75; three-strain: 79). Two deaths (single-strain: 1; three-strain: 1) and one case of NEC (single-strain) that occurred after reaching full feeds were included. Deaths (*n* = 20) that occurred before reaching full feeds and one control with jejunal atresia were excluded from analysis.

(a) *GR volumes (GRV)*: Daily median, maximum, and total GRVs as a percentage of feed volumes (FV) for the period between starting and reaching full feeds (150 mL/kg day) were comparable between groups. Median daily FVs were lower in the control group infants compared with the single- and three-strain group infants in the SiMPro trial (medians: 22.7 vs. 54.8 and 50.9 mL; *p* < 0.001). Similar findings were noted for maximum FVs. The proportion of infants with haemorrhagic residuals was higher in the control vs. SiMPro trial infants (89.7% vs. 32.0% vs. 32.9%, respectively, *p* < 0.001). Median number of haemorrhagic residuals was higher in control vs. SiMPro trial infants (3 vs. 0; *p* < 0.001) (Table 4). (b) *TFF and other outcomes*: Control group infants had longer PN duration (median: 16 vs. 10 days; *p* < 0.001), and were at a higher postnatal age when commenced on minimal enteral feeds (median: 4 vs. 2 days; *p* < 0.001), and reaching full feeds (median: 14 vs. 10 days, *p* = 0.022) compared to SiMPro trial infants. Incidence of NEC ≥ stage II, LOS, and growth parameters were comparable between control group and SiMPro trial infants (Table 2). Analysis adjusting for the IUGR and CRIB score showed that compared to the control group, the TFF was significantly reduced in SiMPro trial infants (adjusted main effects: 0.63, 95% CI 0.48–0.83, *p* = 0.001 and 0.67, 95% CI 0.52–0.87, *p* = 0.003 in the single- and three-strain groups, respectively).

The duration of PN, age at commencing minimal enteral feeds, and TFF were comparable after excluding infants with IUGR (Controls: 16; single-strain SiMPro: 70; three-strain SiMPro: 75) (Supplementary Table S2).

## 4. Discussion

Our results showed that the bile acid content was higher in dark vs. pale green GRs (825.79 vs. 338.1 µmol/L), although it was statistically not significant. There were no significant differences in the nitrogen, carbohydrate, and fat content of pale vs. dark green GRs in SiMPro trial infants. Overall mean GR loss of fat, nitrogen, and carbohydrate over the study period was 0.02 g, 0.011 g, and 0.05 g, respectively.

Bile-stained, especially dark green GRs are often considered as a marker of feed intolerance (? early NEC) and discarded. Variability in interpretation of colored GRs can impact on decisions related to feeding [35,36]. Mihatsch et al. showed that isolated green GRs in the absence of other clinical signs were not negatively correlated with feeding volume on day 14 in preterm infants, and that isolated green GRs should not slow the advancement of feeds [37]. Bertino et al. reported that hemorrhagic and not green GRs were better predictors of NEC [38]. Considering these contradictory conclusions, the rationale for discarding green GRs could be questioned, especially in the context of the physiological role of bile.

The important functions of bile acids include nutrient absorption and metabolism, gut motility, and protection of gut mucosa from pathogens. Bile acids enhance nutrient absorption by acting as signaling molecules and activating bile acid-activated receptors (e.g., farnesoid-X-receptor (FXR), G protein-coupled bile acid receptor 1 (GPBAR1/TGR5)). Both FXR and GPBAR1 help in maintaining the tolerogenic state of the hepatic and intestinal components of innate immunity and modulating gut microbiota [20,25,26,39,40,41]. FXR activation is involved in lipid, glucose, and drug metabolism [42], as well as epithelial cell proliferation [43]. TGR5 is predominantly expressed in the enteric nervous system and influences distal gut motility [44,45]. Bile acids are known to stimulate intestinal motor activity [22,46,47]. Motilin released into circulation by biliary output induces Phase 3 of migratory motor complexes, which promote absorption of bile acids in the distal intestine [21]. Furthermore, reduced bile acid levels in the gut are associated with bacterial overgrowth and inflammation [48]. Bile salts and gut microbiota share an intricate relationship. On one hand, intestinal bile salt structure is influenced by bacterial metabolism; on the other hand, the size and composition of the gut microbiota (and hence intestinal homeostasis) are affected by bile salts [49,50]. The gut microbiota uses bile salts as environmental signals, nutrients, and electron acceptors. The antibacterial effects of bile salts relates to disruption of bacterial membranes, denaturation of proteins, chelation of iron and calcium, oxidative DNA damage, and control of gene expressions involved in host defense and immunity [50]. In the proximal small intestine, bile acids protect gut mucosa against pathogens by their amphipathic property, and solubilizing ability. In the distal small intestine, this protection is mediated by increased synthesis and secretion of antimicrobial factors from intestinal epithelium by gene induction through interaction with FXR [39]. Given these data, the practice of discarding isolated green GRs containing bile acids represents a potential barrier to optimizing enteral nutrition in early postnatal life in EP infants when optimizing enteral feeding is a priority.

The findings of reduced TFF in infants in the SiMPro trial (all received probiotic) vs. control group (no probiotic) need to be discussed. Compared to the control group, the TFF was significantly reduced in SiMPro trial infants (median 14 vs. 10 days) irrespective of their allocation to single- or three-strain probiotics. The higher median FVs and comparable GRVs in SiMPro vs. control group infants suggest the beneficial effects of probiotics on gastric emptying and gut motility [28,29].

To our knowledge, this is perhaps the first study assessing the composition of bile-stained GRs. The inclusion of EP infants from double-blind RCTs of probiotic supplementation optimizes the validity of our findings despite the small sample size. This approach also helped us in addressing the confounders related to GRs [51,52]. The duration of our study covers the critical early postnatal period when EP infants are at high risk of nutritional deprivation. Furthermore, our methodology for assessing composition of GRs is sound, and we adjusted our analyses to control for imbalance in baseline characteristics, such as CRIB score and IUGR. The limitations of our study include the loss of 29 samples from the SiMPro cohort due to inadequate volume for analysis. Ideally, we should have recruited all infants from the PANTS and SiMPro trial. Our study was not powered to detect significant changes in NEC ≥ Stage II as an important outcome. Furthermore, caution is required in interpreting the results of our post hoc analysis of secondary outcomes despite the use of regression analyses. This is because of the small numbers and the risk of selection bias with regard to the reference group of infants.

## 5. Conclusions

In summary, our results show that discarding dark green GRs could result in significant loss of bile acids, an important nutrient, during early enteral nutrition in EP infants. They provide further evidence to question the practice of routine monitoring and interpretation of GRs in preterm infants [12]. A Cochrane systematic review [53] assessed the safety and efficacy of re-feeding or discarding GRs in preterm infants, and concluded that limited data from one small unblinded trial (*n* = 72) [54] with overall low to very low quality of evidence is insufficient to support or refute re-feeding of GRs in preterm infants.

## Figures and Tables

**Table 1:** Neonatal and maternal characteristics.

Characteristics	Control ^a^ *n* = 29	Single-Strain ^b^ *n* = 75	Three-Strain ^c^ *n* = 79	*p*-Value
Gestational age (w) *	26.1 (25.2–26.9)	26.3 (24.7–27.3)	26.6 (25.0–27.3)	0.684
Male ^#^	13 (44.8%)	42 (56.0%)	40 (50.6%)	0.566
Birthweight (g) *	810 (685–970)	870 (708–1010)	920 (750–1070)	0.145
IUGR ^#^	13 (44.8%)	5 (6.7%)	4 (5.1%)	<0.001
Caesarean section ^#^	13 (44.8%)	48 (64.0%)	43 (54.4%)	0.117
Apgar < 7 at 5 min ^#^	9 (31.0%)	21 (28.0%)	20 (25.3%)	0.828
CRIB score ^	10 (3–12)	12 (9–14)	10 (9–13)	0.009

Data represent: * median, interquartile range; ^#^: number and percentage ^Mean and Standard Deviation (SD). ^a^: Infants from the placebo arm of PANTS RCT, ^b^: Infants from the single-strain probiotic arm of SiMPro RCT, ^c^: Infants from the three-strain probiotic arm of the SiMPro RCT. Abbreviations: CRIB-clinical risk index for babies, IUGR-intrauterine growth restricted.

**Table 2:** Nutritional outcomes and growth at discharge.

Outcomes	Control ^a^	Single Strain ^b^	Three-Strain ^c^	*p*-Value
PN duration (days) *	16 (13–22)	10 (7–13)	10 (8–16)	<0.001
Age probiotic/placebo started (days) *	7 (5–10)	3 (2–4)	3 (2–4)	<0.001
Age MEF started (days) *	4 (3–7)	2 (2–3)	2 (2–3)	<0.001
TFF (days) *	14 (12–20)	10 (8–15)	10 (7–16)	0.022
EBM ^#^	23 (79.3%)	66 (88%)	72 (91.1%)	0.222
PDHM ^#^	6 (20.7%)	28 (37.3%)	25 (31.6%)	0.263
NEC ≥ stage II ^#^	0 (-)	1 (1.3%)	0 (-)	0.568
LOS ^#^	7 (24.1%)	18 (24.0%)	13 (16.5%)	0.472
Weight z-score at discharge ^	−0.64 (1.82)	−0.79 (0.90)	−0.71 (0.98)	0.824
Length z-score at discharge ^	−1.07 (2.33)	−1.27 (1.76)	−0.88 (1.36)	0.390
HC z-score at discharge ^	−0.32 (1.74)	0.06 (1.48)	−0.15 (1.72)	0.519

Data represent: * median, interquartile range, ^#^ number and percentage; ^: Mean and Standard Deviation (SD). ^a^: Infants from the placebo arm of PANTS RCT, ^b^: Infants from the single-strain probiotic arm of SiMPro RCT, ^c^: Infants from the three-strain probiotic arm of the SiMPro RCT. Abbreviations: CRIB-clinical risk index for babies, EBM-Expressed Breast Milk, IUGR-intrauterine growth restricted, LOS-Late Onset Sepsis (culture proven), MEF-Minimal Enteral Feeds, NEC-Necrotising Enterocolitis, PDHM-Pasteurised Donor Human Milk, PN-Parenteral Nutrition

**Table 3:** Analysis of Gastric Residual compared between pale and dark green colour grades.

Nutrients in GR Title	Mean Estimates (95% CI)	Mean Difference (95% CI)	*p*-Value
Bile acid ^@^ (µmole/L)			
Pale green	338.10 (126.47–903.84)	reference	
Dark green	825.79 (469.84–1451.40)	2.44 (0.78–7.62)	0.120
pH			
Pale green	3.83 (2.68–4.97)	reference	
Dark green	4.25 (3.49–5.02)	0.43 (−0.94–1.80)	0.532
Osmolality ^@^ m(OsM)			
Pale green	342.71 (318.78–368.41)	reference	
Dark green	357.71 (338.24–378.29)	1.04 (0.95–1.15)	0.356
Fat content ^@^ (g/L)			
Pale green	14.02 (7.52–26.13)	reference	
Dark green	13.03 (8.96–18.95)	0.93 (0.47–1.83)	0.829
Overall loss (g)	0.02 (0.01–0.03)		
Total nitrogen ^@^(g/L)			
Pale green	7.08 (5.63–8.90)	reference	
Dark green	8.49 (7.20–10.01)	1.19 (0.93–1.55)	0.161
Overall loss (g)	0.011 (0.009–0.013)		
CHO ^@^ (g/L)			
Pale green	34.19 (25.35–46.12)	reference	
Dark green	36.80 (28.42–47.65)	1.08 (0.72–1.60)	0.711
Overall loss (g)	0.05 (0.04–0.06)		

^@^ Estimated geometric means and differences and 95% confidence intervals are reported. The back transformed mean differences for these estimates represent the proportion change from the reference group. A confidence interval including one is not statistically significant. GR-Green gastric residuals.

**Table 4:** Summary of daily Gastric Residual Volumes (GRV), feed volumes (FV), and colour of aspirate from commencement of minimal enteral feeds until reaching 150 mL/kg/day.

GRV, FV and Coloured/ Hemorrhagic GRs	Control ^a^ *n* = 29	Single-Strain ^b^ *n* = 75	Three-Strain ^c^ *n* = 79	*p*-Value
GRV as a % of FV				
Median	4.4 (3.0–7.4)	4.9 (2.1–6.9)	4.5 (2.0–9.6)	0.899
Maximum	67.5 (26.6–159)	50.5 (25.0–100.0)	70.0 (23.3–160.0)	0.324
Total	168.5 (111.8–372.9)	152.0 (72.5–249.8)	240.7 (57.2–358.5)	0.267
GRV (mL)				
Median	1.5 (0.9–2.1)	2.3 (0.8–3.5)	2.0 (1.2–4.0)	0.122
Maximum	7.5 (5.7–13.3)	10.5 (7.0–16.7)	11.0 (6.9–17.0)	0.160
Total	38.0 (21.8–51.6)	34.8 (19.5–70.9)	37.5 (21.8–76.0)	0.857
FV (mL)				
Median	22.7 (13.9–50.0)	54.8 (34.0–76.0)	50.9 (28.0–72.0)	<0.001
Maximum	138.0 (110.5–156.5)	144.0 (114.5–163.0)	155.0 (121.0–176.0)	0.009
Total	696.5 (489.5–935.5)	620.0 (471.9–1001.8)	660.0 (514.0–1106.5)	0.746
Any haemorrhagic residuals	26 (89.7%)	24 (32.0%)	26 (32.9%)	<0.001
Number of haemorrhagic residuals	3 (1.5–5.5)	0 (0–1)	0 (0–1)	<0.001
Any coloured residual	25 (86.2%)	57 (76.0%)	62 (78.5%)	0.521
Number of coloured residuals	4 (1.5–6)	3 (1–4)	2 (1–5)	0.238

Data represents: median, interquartile range, number (%), as appropriate. ^a^: Infants from the placebo arm of PANTS trial, ^b^: Infants from the single-strain probiotic arm of SiMPro trial, ^c^: Infants from the three- strain probiotic arm of the SiMPro trial.

## Supplementary Materials

The following are available online at https://www.mdpi.com/2072-6643/12/9/2585/s1, Table S1. (a): Raw values of aspirate characteristics for the total sample and among samples stratified by colour grade; (b): Raw values of aspirate characteristics among samples stratified by SiMPro treatment groups, Table S2. Nutrition and feeding
