# Supplementary material for: Composition of Coloured Gastric Residuals in Extremely Preterm Infants-A Nested Prospective Observational Study
Source: Nutrients. 2020 Aug 26;12(9):2585. doi: 10.3390/nu12092585 (PMC7551671; doi:10.3390/nu12092585)
Supplement: Supplementary file 1 [file nutrients-12-02585-s001.pdf]

Table S1. (a): Raw values of aspirate characteristics for the total sample and among samples stratified by colour grade; (b): Raw values of aspirate characteristics among samples stratified by SiMPro treatment groups.

(a)

Characteristics of Aspirate	Total Sample *n* = 74	Pale Green *n* = 15	Avocado *n* = 30	Spinach *n* = 29
Volume (g)	1.26 (0.89–2.13)	1.44 (1.00–2.43)	1.63 (1.01–2.81)	1.12 (0.63–1.30)
pH	4.31 (2.88–5.23)	4.23 (2.72–4.90)	3.74 (2.81–4.74)	4.87 (3.05–6.05)
Osmolality (mOsM)	332 (311–373)	324 (305–368)	333 (315–413)	347 (306–373)
Nitrogen (g/L)	7.72 (5.75–10.25)	7.05 (5.18–9.42)	8.42 (5.72–11.51)	7.63 (6.10–11.84)
CHO (g/L)	38.36 (22.48–73.87)	35.03 (23.93–56.93)	40.38 (24.13–80.43)	30.05 (10.70–80.21)
Bile acid (µmole/L)	1322.22 (369.45–2492.65) *n* = 52	270.84 (101.04–1258.34) *n* = 8	1388.89 (544.45–2014.71) *n* = 17	1388.89 (533.33–2617.65) *n* = 27
Fat content (g/L)	17.49 (6.81–25.87) *n* = 71	19.96 (6.87–25.87) *n* = 15	20.68 (10.90–32.92) *n* = 28	10.38 (5.13–18.27) *n* = 28
**Treatment group**				
Single-strain	32 (43.2%)	7 (46.7%)	15 (50.0%)	10 (34.5%)
Three-strain	42 (56.8%)	8 (53.3%)	15 (50.0%)	19 (65.5%)

Data represent median and interquartile range.

(b)

Characteristics of Aspirate	Single-strain Samples *n* = 32	Three-strain Samples *n* = 42
Volume (g)	1.29 (0.90–2.09)	1.25 (0.86–2.21)
pH	3.77 (2.79–4.91)	4.55 (3.23–5.65)
Osmolality m(OsM)	348 (314–374)	325 (309–372)
Nitrogen (g/L)	7.62 (5.37–9.91)	7.80 (6.04–10.84)
CHO (g/L)	43.21 (19.24–79.52)	36.02 (22.48–63.17)
Bile acid (µmole/L)	1322 (742–2574) *n* = 22	1250 (133–2360) *n* = 30
Fat content (g/L)	19.83 (9.68–26.07) *n* = 31	14.68 (5.13–21.52) *n* = 40

Data represent median and interquartile range.

Table S2. Nutrition and feeding.

	Control *n* = 16	Single-strain *n* = 70	Three-strain *n* = 75	*p*-value
TPN duration[@]	16 (13–20)	10 (7–13)	10 (8–16)	0.001
Age (d) reached minimal enteral feeds[@]	4 (3–7)	2 (2–3)	2 (2–3)	< 0.001
EBM[#]	15 (93.8%)	58 (82.9%)	57 (76.0%)	0.239
PDHM[#]	1 (6.3%)	21 (30.0%)	31 (41.3%)	0.020
Age (d) reached 150 mL/kg/day/ TFEF[@]	18 (15–24)	13 (10–18)	12 (10–20)	0.008
EBM[#]	15 (93.8%)	61 (87.1%)	68 (90.7%)	0.698
PDHM[#]	1 (6.3%)	28 (40.0%)	25 (33.3%)	0.263
Time to full feeds (day)[@]	14 (11–18)	10 (8–16)	10 (7–16)	0.152

Data represent: @: median, interquartile range, #: *n* (%), as appropriate. Abbreviations: PN-parenteral nutrition, EBM-expressed breast milk, PDHM-pasteurised donor human milk.
